# Profiling of adenine‐derived signaling molecules, cytokinins, in myotubes reveals fluctuations in response to lipopolysaccharide‐induced cell stress

**DOI:** 10.14814/phy2.15870

**Published:** 2023-12-01

**Authors:** Stephanie W. Tobin, Dev Seneviratne, Lorna Phan, Mark Seegobin, Alexander L. Rico, Beth Westby, Anna Kisiala, Sanela Martic, Craig R. Brunetti, R. J. Neil Emery

**Affiliations:** ^1^ Department of Biology Trent University Peterborough Ontario Canada; ^2^ Environmental and Life Sciences Graduate Program Trent University Peterborough Ontario Canada; ^3^ Department of Forensic Science Trent University Peterborough Canada

**Keywords:** cytokinin, muscle, myokine

## Abstract

Cytokinins (CTKs) are a diverse collection of evolutionarily conserved adenine‐derived signaling molecules classically studied as phytohormones; however, their roles and production have been less studied in mammalian systems. Skeletal muscles are sensitive to cellular cues such as inflammation and in response, alter their secretome to regulate the muscle stem cell and myofiber niche. Using cultured C2C12 muscle cells, we profiled CTK levels to understand (1) whether CTKs are part of the muscle secretome and (2) whether CTKs are responsive to cellular stress. To induce cellular stress, C2C12 myotubes were treated with lipopolysaccharides (LPS) for 24 h and then media and cell fractions were collected for ultra high‐performance liquid chromatography tandem mass spectrometry with electrospray ionization (UHPLC‐(ESI+)‐HRMS/MS) for metabolomics and CTK profiling. Across LPS‐treated and control cells, 11 CTKs were detected in the extracellular space while 6 were detected intracellularly. We found that muscle cells are enriched in isopentenyladenine (iP) species (from free base, riboside to nucleotide forms), and that extracellular levels are increased after LPS treatment. Our study establishes that muscle cells express various forms of CTKs, and that CTK levels are responsive to LPS‐induced cell stress, suggesting a role for CTKs in intra‐ and extracellular signaling of mammalian cells.

## Graphical abstract

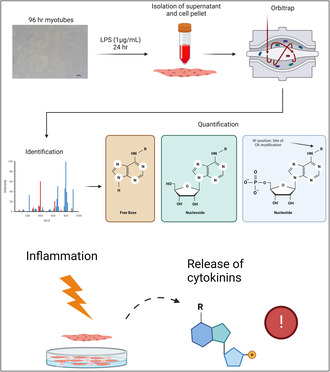

## 1 INTRODUCTION

Cytokinins (CTKs) are a class of powerful signaling molecules most known for their role in plants and cytokinesis but are now recognized to have much broader biological roles across taxa, from bacteria to mammals. CTKs are adenine derivatives and possess either an isoprenoid or aromatic side chain at the N^6^‐position which determines the CTK species and what properties they exhibit (Hluska et al., 2021). Plant studies show that CTKs can have various biological activities depending on their form (Hluska et al., 2021), here organized from least bioactive to most highly bioactive: nucleotides, ribosides/nucleosides, and free bases/nucleobases. Activity of these structures appears to be quite different in mammals wherein the NT forms are more bioactive and act as substrates for reactions. For example, it is the NT forms of both kinetin (kinetin triphosphate) and N^6^‐isopentenyladenosine‐5'mono/di/triphosphate (iPNT) that are active in DNA repair (Bowie et al., 2018) or AMP‐activated protein kinase (AMPK) activation (Pisanti et al., 2014), respectively. Additionally, given that CTKs are adenine derivatives, purinergic signaling (i.e., the use of ATP, ADP, AMP, or adenosine as extracellular signaling molecules) is of particular biological importance.

Kinetin and N^6^‐(isopentenyl)adenine (iP) species have drawn attention for their activity in mammalian systems; the former in Huntington's disease (Bowie et al., 2018), myogenesis (Mielcarek & Isalan, 2021), aging (Sharma et al., 1995), and both in cancer (Choi et al., 2008; Ishii et al., 2002; Laezza et al., 2009; Laezza et al., 2010; Voller et al., 2019). In C2C12 myoblasts, exogenous kinetin promotes myogenesis and glucose uptake, and functions as an antioxidant (Mielcarek & Isalan, 2021). These two examples of CTKs (kinetin and iP) fall into two groups of interest important for the scope of this study: (1) CTKs generated by DNA degradation (e.g., N^6^‐furfuryladenine also called kinetin [Barciszewski et al., 2000]) and (2) CTKs generated by canonical pathways known from plants: the de novo adenylate pathway (e.g., iP, trans‐Zeatin [tZ], dihydrozeatin [DZ]), and the tRNA‐degradation pathway (e.g., iP, cis‐Zeatin [cZ], and 2MeS species). In mammals, the tRNA‐degradation pathway serves as the primary source of CTKs where they are first synthesized as A37 modifications on a subset of tRNAs, and subsequently alter tRNA stability, interactions with the ribosome, and translation efficiency (Horvath & Chinnery, 2015; Jenner et al., 2010). The triggers of DNA or tRNA degradation that release these CTKs are unclear, and while most studies in mammalian systems have focused on exogenous applications, there are a few reports that analyze levels of endogenous CTKs (Aoki et al., 2019; Barciszewski et al., 1996; Barciszewski et al., 2000; Seegobin et al., 2018).

While it is established that CTKs are indeed expressed in a wide variety of whole tissues from mammals, including canine muscles (Seegobin et al., 2018), we do not know whether endogenous CTK levels may change in muscles in response to cellular stress. In this study we characterized CTK species and levels in cultured myotubes treated with lipopolysaccharides (LPS) as a method to induce cellular stress and catabolism (Ono & Sakamoto, 2017). Myotubes were chosen for this study for two reasons: (1) Myotubes demonstrate robust and established programs of cellular stress, with well‐documented catabolic programs activated after treatment with LPS. (2) In vivo, skeletal muscle has an active role in maintaining its local microenvironment, which is composed of a mix of muscle stem cells (MuSCs), fibro/adipogenic progenitors (FAPs), and tissue‐resident immune cells, through paracrine signaling to regulate homeostatic and regenerative processes (Bentzinger et al., 2013; Biferali et al., 2019; Hindi & Millay, 2021; Relaix et al., 2021). Here, we report that CTKs are indeed expressed in cultured muscle cells and that in response to LPS, there is a dynamic change in CTK species, predominately the iP species. Notably, the response of extracellular CTKs was more pronounced than intracellular CTKs, and included a mixture of all forms of iP, from free base, nucleoside, and nucleotide.

## 2 METHODS

### 2.1 Cell culture for cytokinin and metabolite isolation

C2C12 cells from the American Tissue Culture Collection (ATCC) were grown in Dulbecco's Modified Eagle Medium (DMEM) with high glucose (Millipore Sigma, Oakville, Ontario, Canada, Cat no. D6429) supplemented with 10% fetal bovine serum (FBS) (Cytivia Hyclone, Fisher Scientific, Whitby, Ontario, Canada, Cat no. SH3039603HI) and 1% Penicillin–Streptomycin (P/S; Invitrogen, Burlington, Ontario, Canada, Cat no. 15140122). When cells were approximately 90% confluent, medium was replaced with differentiation medium (DM) containing DMEM supplemented with 2% horse serum (Cytivia Hyclone, Fisher Scientific, Cat no, SH3007403) and 1% P/S. Cells were maintained in a humidified 37°C incubator at 5% CO_2_. After 96 h in DM, cells were washed twice with phosphate buffered saline (PBS) and lipopolysaccharides from *Escherichia coli* (LPS, O55:B5, Millipore Sigma, Cat no. L6529) were added to fresh serum‐free DMEM (plus 1% P/S) or fresh DM at a final concentration of 1 μg/mL for 24 h.

### 2.2 Cell counting kit 8 for cell viability

C2C12 cells were seeded into 96‐well plates. When the cells were 95% confluent, growth medium was changed to low serum DM for 96 h, with fresh media added every 48 h. After 96 h in DM, fresh serum‐free DMEM was applied with LPS at a concentration of 0.1–10 μg/mL for 24 h. To determine cell viability, cells were incubated with 10 μL of cell counting kit 8 (CCK‐8/WST‐8, Abcam, Cambridge, MA, USA, Cat no. ab228554) reagent for 2 h, according to the manufacture's instructions. Absorbance was measured at 460 nm. Readings were normalized to blank wells.

### 2.3 Hydrogen peroxide/peroxidase detection kit

Myotubes were prepared as described above and treated with LPS in serum‐free DMEM (plus 1% P/S) at a final concentration range 0.1–10 μg/mL for 24 h. Conditioned media was collected from each condition and used with the Fluoro H_2_O_2_™ Hydrogen Peroxide/Peroxidase Detection Kit (Cell Technology, Mountain View, CA, USA, Cat no. FLOH100‐3) to determine levels of H_2_O_2_ in response to LPS treatment.

### 2.4 Preparation of samples for CTK analysis and metabolite expression

Each replicate (*n* = 3 from serum‐free DMEM, *n* = 3 from 2% HS) was derived from a 100 mm culture dish of differentiated cells as described above. To evaluate extracellular CTK and metabolite content, 10 mL supernatant was collected for each replicate and lyophilized. In addition, whole cell lysates were collected in PBS using a cell scraper and pelleted via centrifugation (200 × g for 5 min). Samples were stored at −80°C prior to metabolite extraction. Samples were resuspended in 1 mL of ice‐cold 50% acetonitrile (CAN) before they were spiked with stable‐isotope labeled canonical amino acids (0.25 μM; Cambridge Isotope Laboratories, Tewksbury, MA, USA) and 10 ng of deuterated internal standards (OlChemim Ltd. [Olomouc, Czech Republic]) corresponding to the different forms of CTKs (Aoki et al., 2021; Bean et al., 2022; Kisiala et al., 2019; Palberg et al., 2022). Subsequently, samples were homogenized and then allowed to passively extract overnight at ‐20°C. Extracts were collected following centrifugation (11,180 × g for 10 min) and purified by solid phase extraction (SPE) using HLB cartridges as previously described (Šimura et al., 2018). Following elution in 30% CAN, 1 mL was aliquoted for untargeted metabolomics and the remaining 3 mL was used for CTK‐targeted scans. Aliquots were evaporated in a speed vacuum concentrator (Savant SPD111V, UVS400, Thermo Fisher Scientific) at room temperature. Samples designated for untargeted analysis were reconstituted in 500 μL of 90% CAN and stored at ‐20°C.

Extraction and purification of CTKs were performed as previously described (Kisiala et al., 2019). Samples were reconstituted in 1 mL 1 M formic acid and processed via SPE on a mixed‐mode, reversed‐phase, cation‐exchange cartridge (MCX 6 cc, 200 mg; Canadian Life Sciences, Peterborough, Ontario, Canada). Cartridges were conditioned with 5 mL of methanol and formic acid before sample extracts were loaded and washed with 5 mL of formic acid and methanol. CTK nucleotides were eluted with 5 mL 0.35 M ammonium hydroxide (NH_4_OH) while free base, riboside, and methylthiol species were subsequently eluted together using 5 mL of 0.35 M ammonium hydroxide in 60% methanol. Fractions were evaporated in a speed vacuum concentrator at room temperature and stored at −20°C until further processing. As our current LC–MS/MS method cannot directly analyze CTK nucleotides, these fractions were dephosphorylated using 3 units calf intestine alkaline phosphatase (CIP; New England BioLabs, Whitby, Canada) in 1 mL of 0.1 M ethanolamine hydrochloride overnight at 37°C before evaporation at room temperature. The residues were reconstituted in 1.5 mL B‐Pure water prior to purification on C_18_ cartridges (C_18_ 6 cc, 500 mg; Canadian Life Sciences, Peterborough, ON, Canada). Cartridges were conditioned with 3 mL of methanol and 6 mL of B‐Pure water before the samples were loaded and washed with 3 mL B‐Pure water. The ribosides were eluted using 1.25 mL 80% methanol, evaporated and stored at −20°C until further processing. In preparation for analysis, all extracts were reconstituted in 500 μL starting LC mobile phase (water‐acetonitrile [95:5], v/v, with 0.08% glacial acetic acid).

### 2.5 CTK quantification using ultra high‐performance liquid chromatography‐positive electrospray ionization‐high resolution tandem mass spectrometry (UHPLC‐(ESI+)‐HRMS/MS)

Endogenous CTKs were identified and quantified using UHPLC‐(ESI+)‐HRMS/MS following a previous protocol optimized for small molecules (Kisiala et al., 2019). A 25 μL sample volume was injected into the Thermo Ultimate 3000 UHPLC coupled to a Thermo Q‐Exactive™ Orbitrap mass spectrometer equipped with a heated electrospray ionization (HESI) source (Thermo Scientific, San Jose, CA, USA). Compounds were separated using a reversed‐phase C18 column (Kinetex 2.6 μ C18 100 A, 2.1 × 50 mm; Phenomenex, Torrance, CA, USA). CTK species were eluted with a multistep gradient of water (A) and acetonitrile (B) both mixed with 0.08% glacial acetic acid at a flow rate of 0.5 mL/min. Initially, 5% B was held for 0.5 min, increasing linearly to 45% B over 4.5 min followed by an increase to 95% B over 0.1 min; and held at 95% B for 1 min, before returning to initial conditions for 2 min of column re‐equilibration, in a total run time was 8.2 min. Cytokinins were analyzed using parallel reaction monitoring (PRM) at 35,000 resolution; all samples were analyzed in positive ion mode. The HESI‐II auxiliary gas heater and capillary were operated at 450 and 300°C, respectively, sheath gas at 30 (arbitrary units), auxiliary gas at 8 (arbitrary units), sweep gas at 0 (arbitrary units), S‐lens RF level 60, and spray voltage at 3.9 kV. Additional PRM parameters included automatic gain control (AGC) of 3 × 10^6^, maximum injection time of 128 ms, m/z 1.2 isolation window, and normalized collision energy individually optimized for each analyte. CTK levels were calculated as pmol retained in cell pellet or released (supernatant) per gram of fresh weight of myotube cells (pmol/gFW).

All data were analyzed using Thermo Xcalibur (v. 4.1) software (Thermo Scientific, San Jose, CA, USA), to calculate peak areas. Quantification of CTKs was achieved through isotope dilution analysis based on recovery of ^2^H‐labeled internal standards. Blank media samples (control, MAM solution) were processed and analyzed using the same methodologies.

### 2.6 Metabolomics

The fraction of sample eluates collected from HLB cartridges was used for untargeted metabolomics analysis. The metabolomic data were acquired in full scan (FS) mode using the high‐resolution Thermo Q‐Exactive™ Orbitrap mass spectrometer. The samples were injected into the mass spectrometer through the Thermo Ultimate 3000 HPLC system using the parameters for small molecules as previously mentioned. Regarding FS analysis, each sample was run simultaneously in positive and negative ion mode over the mass range of m/z 70–900, at 70,000 resolution, with an ACG target of 1 × 10^6^, and a maximum injection time of 100 ms.

Full scan and ddMS2 data were processed in Xcalibur 4.1 software using a quantification method for metabolites related to cell metabolism. Metabolites were identified at two confidence levels according to Schrimpe‐Rutledge et al (2016); Level 1, by mass (within 10 ppm accuracy) and comparison of retention times to authentic labeled (amino acids) and unlabeled, high purity standards for HPLC analysis, or Level 2, by mass and comparison of fragment spectra (authentic standards, METLIN, PubChem databases). Normalized metabolite levels were calculated based on the median recovery of the labeled internal standards (amino acids) in each sample. Relative metabolite levels were then normalized to the maximum detection of each metabolite across all samples.

### 2.7 Data analysis

All statistical analyses were performed using GraphPad Prism v 10. Pairwise comparisons were performed using multiple unpaired *t*‐tests. To compare cellular responses to increasing concentrations of LPS, data were analyzed using a one‐way ANOVA followed by Tukey's post hoc analysis. Data are presented as mean ± SD, *n* = 3–6.

## 3 RESULTS

### 3.1 Cytokinins typical of the de novo and tRNA degradation pathway are detected in C2C12 myotubes

**FIGURE 1:**
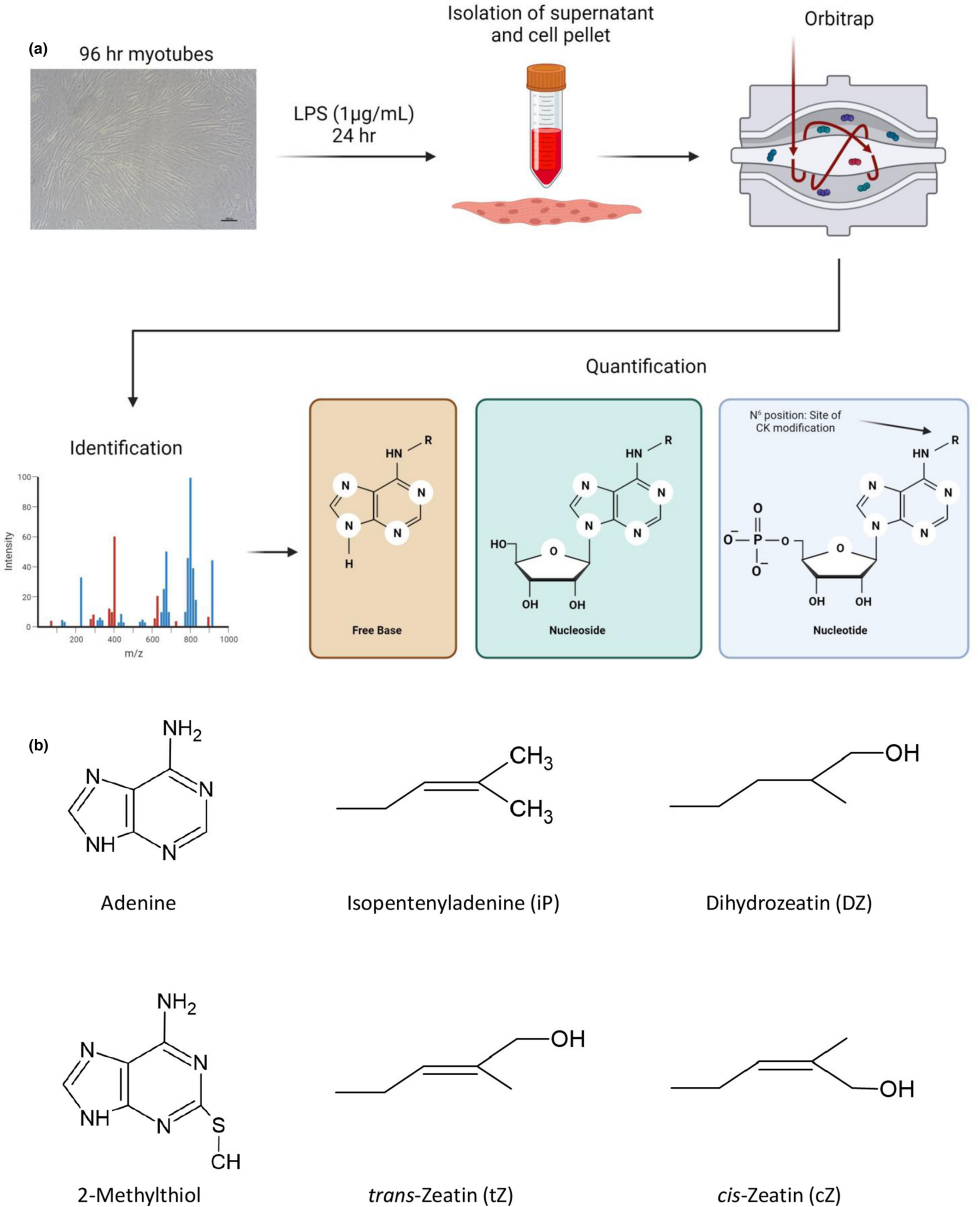
Cytokinin profiling and metabolomics of C2C12 myogenic cells. **(**a) C2C12 myoblasts were differentiated to myotubes in low serum differentiation medium (2% horse serum) for 96 h followed by 24 h treatment with lipopolysaccharides (LPS, 1 μg/mL). Cell pellets and supernatants were collected for intracellular and extracellular assessment of cytokinin (CTK) levels via HPLC‐(HRAM)MS/MS. Metabolomics was also completed on the cell fraction. Over 30 CTKs and 90 metabolites were screened, and 11 and 86 were detected and quantified, respectively. Types of CTKs screened included the free base, nucleoside, and nucleotide forms. Note that the CTK modification occurs at the N^6^ position, notated as “R” in the cartoon. Image was created with Biorender.com. (b) Chemical structures representing CTKs detected. Structures represent sidechains present at either the N^6^ position or the addition of a methylthiol group at position 2 of the adenine ring. Image was created with ChemSketch.

To establish a CTK profile in response to cellular stress, we used the C2C12 myoblast cell line and collected medium and cell pellets from differentiated myotubes treated for 24 h with lipopolysaccharides (LPS) and from control cultures (Figure 1). LPS activates MAP kinases JNK and p38, impairs mitochondrial function and translation, and increases protein ubiquitination and autophagy (Frisard et al., 2015; McClung et al., 2010; Puigserver et al., 2001) and is therefore a robust activator of cellular catabolism. Our previous study showed that CTK levels are minimal in DMEM with or without 10% FBS compared to other types of growth media (Aoki et al., 2021) but the composition of CTKs in DMEM supplemented with 2% horse serum has never been evaluated. We therefore prepared equal samples of cells treated with LPS in serum‐free conditions and in 2% horse serum and saw no difference in CTK or metabolite levels (Table S1). Thus, biological replicates were derived from both serum‐free media and media supplemented with 2% horse serum. We scanned for over 30 species of CTKs that differ in their biosynthesis pathway, chemical structure, and known biological activity (Kisiala et al., 2019). Of those, 11 CTKs were detected across LPS‐treated and control cultures: Free base: *N* ^6^‐isopentenyladenine (iP), ribosides: *N* ^6^‐isoepntenyladenosine (iPR), dihydrozeatin‐9‐riboside (DZR), nucleotides (NT): *N* ^6^‐isopentenyladenosine‐5'mono/di/triphosphate (iPNT), dihydrozeatin‐9‐riboside‐5'mono/di/triphosphate (DZNT), trans‐zeatin‐9‐riboside‐5'mono/di/triphosphate (tZNT), cis‐zeatin‐9‐riboside‐5'mono/di/triphosphate (cZNT), and methylthiols (2MeS): 2‐methylythiozeatin (2MeSZ), 2‐methylythiozeatin‐9‐riboside (2MeSZR), 2‐methylthio‐*N* ^6^‐isopentenyladenine (2MeSiP), and 2‐methylthio‐*N* ^6^‐isopentenyladenosine (2MeSiPR).

### 3.2 Metabolite analysis and measures of cellular stress

**FIGURE 2:**
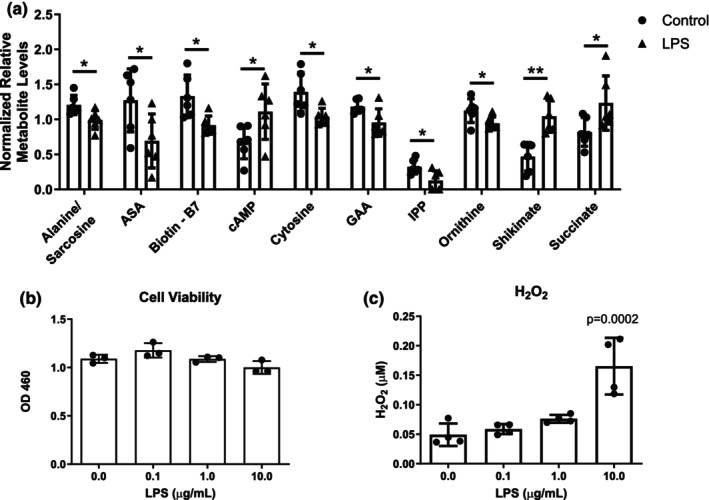
Treatment of myogenic cultures with lipopolysaccharides (LPS) activates programs of inflammation and muscle catabolism but does not cause overt cellular toxicity. (a) Out of a total of 90 metabolites that were identified by mass spectrometry within the cell pellet, 10 were significantly affected by LPS treatment: Adenosine/Sarcosine, acetylsalicylic acid (ASA), biotin—B7, cyclic AMP (cAMP) cytosine, guanidine acetic acid (GAA), isopentenyl pyrophosphate (IPP), ornithine, shikimate, and succinate. Metabolites were analyzed using a two‐tailed unpaired *t*‐test. *p < 0.05, **p < 0.01. (b, c) Cellular toxicity as assessed by the CCK‐8 assay (b, *n* = 3) and hydrogen peroxide (H_2_O_2_) detection (c, *n* = 4). **p < 0.01 based on a one‐way ANOVA and Tukey's post‐hoc analysis. All data are shown as mean ± SD, *n* = 3–6.

To complement the CTK analysis, we utilized full scan mass spectrometry to evaluate over 90 cell metabolites within the cell pellet including amino acids and derivatives, nucleotide metabolites, and constituents of energy metabolism (e.g., sugars, sugar phosphates, and organic acids). Of the 90 features included in the scan, 86 were detected; however, only 10 metabolites showed significant changes in levels in response to LPS treatment (Figure 2a). Three metabolites, cAMP, shikimate, and succinate, were increased after LPS treatment (*p* < 0.05). cAMP and succinate have been shown to increase after LPS treatment in other in vitro cell lines (Bourne et al., 1974; Mills et al., 2016). Shikimate is a secondary metabolite that contributes to the production of aromatic metabolites in plants, bacteria, and fungi and is thought to not be expressed in animals cells, though shikimate‐like molecules do exist (Torres & Schmidt, 2019). Guanidinoacetic acid (GAA) and ornithine feed into the creatine biosynthesis pathway and were reduced by LPS treatment (*p* < 0.05). There was no effect of LPS on levels of branched chain amino acids, adenosine, or AMP. We observed reduced levels of isopentenyl pyrophosphate (IPP, p < 0.05), an isoprenoid precursor (Chang et al., 2013). This is interesting given that some CTKs are categorized based on the presence of an isoprenoid chain.

To better understand the level of cellular stress induced by LPS, we performed two dose response assays: The CCK‐8 assay to test cell viability and a H_2_O_2_ assay to detect the production of reactive oxygen species as a measure of cell stress. Cell viability was not affected by LPS treatment at any tested concentration of LPS, from 0.1 to 10 μg/mL (Figure 2b). We observed a dose‐dependent response in the levels of H_2_O_2_, with a significant increase at 10 μg/mL (Figure 2c, *p* < 0.01). When taken together, the metabolite analysis and CCK‐8 and H_2_O_2_ assays suggest that 24‐h treatment of myotubes with LPS at 1 μg/mL causes cellular stress, but not to the point of cellular toxicity.

### 3.3 LPS treatment increases CTK levels in cultured C2C12 cells

**FIGURE 3:**
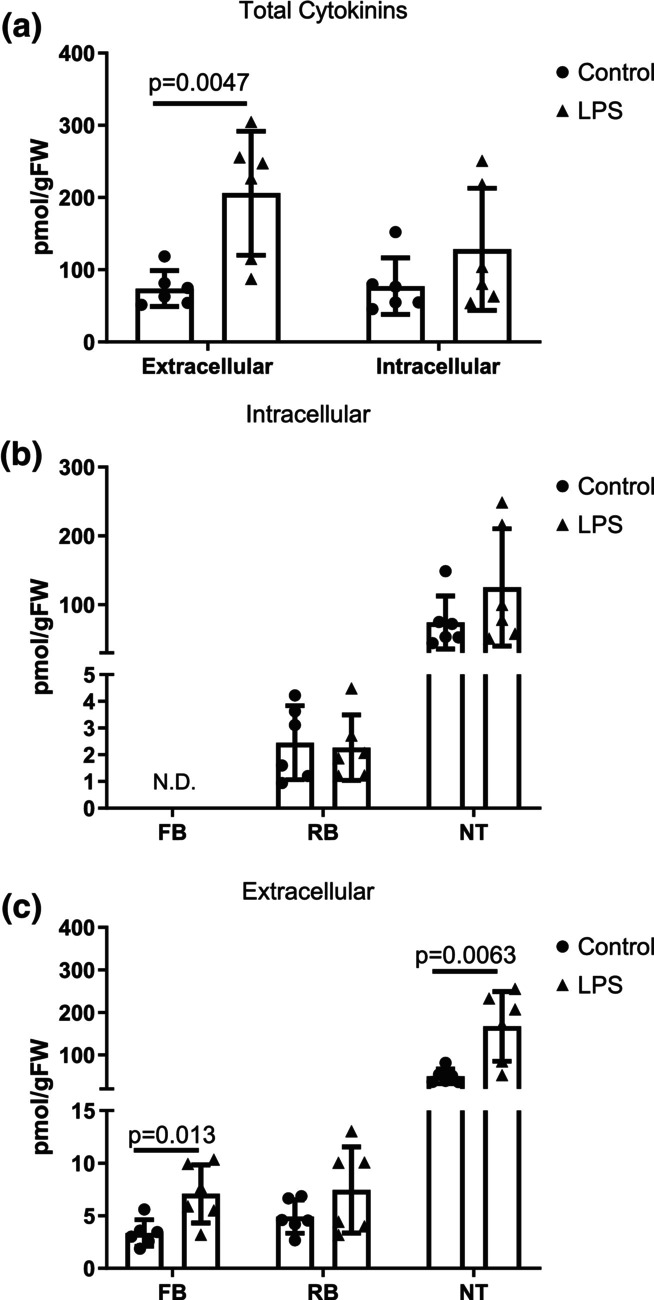
Quantification of total cytokinin (CTK) levels across control and lipopolysaccharide (LPS)‐treated conditions. (a) Total CTKs identified in the cell culture supernatant (extracellular) or cell pellet (intracellular) in response to LPS treatment. (b, c) Types of CTKs present in the extra‐ or intracellular fractions grouped as free base (FB), ribosides (RB), and nucleotides (NT). Data are presented as mean ± SD, *n* = 6. CTK levels were calculated as pmol retained in cell pellet or released (supernatant) per gram of fresh weight of myotube cells (pmol/gFW). Data were analyzed using a two‐tailed unpaired *t*‐test.

While the intra‐ and extracellular spaces contained different CTK species, total CTK content was comparable within control groups (Figure 3a). In response to LPS treatment, however, CTK levels were significantly increased in the extracellular fraction (*p* < 0.01). Next, we considered the total levels of the three forms that CTKs are found in: free bases, ribosides, or nucleotides. In both the extracellular and intracellular compartments, nucleotides were the predominant CTK form detected (Figure 3b,c). Notably, free bases were absent in the cell pellet and both riboside and nucleotide levels were unchanged after LPS treatment (Figure 3b). In contrast, all three forms were detected in the supernatant, with a significant increase in the free bases and nucleotide forms in response to LPS treatment (Figure 3c, *p* < 0.01).

### 3.4 iP species show the most dynamic responses to LPS

**FIGURE 4:**
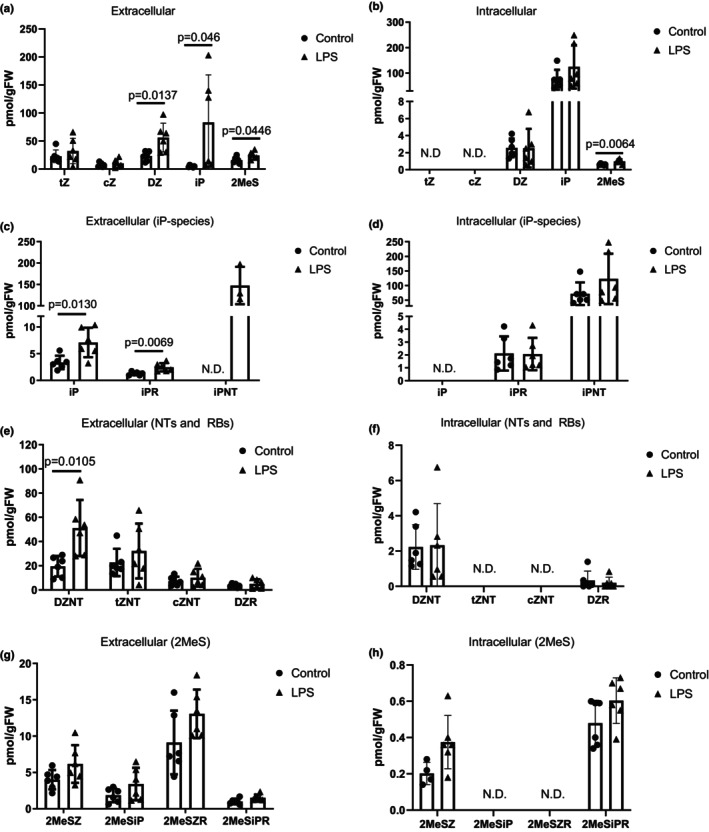
Isopentenyladenine (iP) cytokinins are the most abundant and the most responsive to LPS treatment in myogenic cultures. (a, b) CTK levels as groups based on their family of side chain modification in the extracellular (a) or intracellular fraction (b). tZ: trans‐Zeatin; cZ: cis‐Zeatin; DZ: Dihydrozeatin; N6‐isopentenyladenine (iP); methylthiols (2MeS); ND: not detected. (c, d) Specific profiling of iP as a free base (FB), riboside (RB), or nucleotide (NT) in the extracellular (c) or intracellular fraction (d). (e, f) Specific profiling of detected DZ, tZ, and cZ based on their classification as FBs, RBs, or NT in the extracellular (e) or intracellular space (f). (g, h) Specific profiling of detected methylthiolated forms of Zeatin and iP based on their classification as FBs or RBs in the extracellular (g) or intracellular space (h). CTK levels were calculated as pmol retained in cell pellet or released (supernatant) per gram of fresh weight of myotube cells (pmol/gFW). Data are presented as mean ± SD, *n* = 3–6.

We next pooled the total abundance of all three forms of CTKs (from free base to riboside to nucleotide) based on the type of side chain found at the N^6^ position. trans‐Zeatin (tZ), cis‐Zeatin (cZ), dihydrozeatin (DZ), isopentenyladenine (iP), as well as methylthiolated (2MeS) forms were detected in the supernatant while tZ and cZ CTKs were absent in the cell pellet (Figure 4a,b). 2MeS species were increased in both cellular and acellular fractions in response to LPS, while in the supernatant, levels of DZ and iP species were also increased (*p* < 0.05).

Figure 4c–h provides a further breakdown of the specific CTK forms (e.g., free base vs riboside vs nucleotide forms of tZ, cZ, DZ, iP, and 2MeS). The most striking observation was the consistent increase of iP species that was evoked by LPS in the extracellular space: There was a significant increase in the free base (*p* < 0.05), riboside (*p* < 0.01), and nucleotide forms of iP (Figure 3c). As iPNT was not detected in the extracellular fraction of the control treatment, statistical analysis was not performed; however, there is a clear increase in iPNT levels in response to LPS. These LPS‐induced increases of iP species were not reflected in the cell pellet; however, iPNT was still the most abundant form of any CTK detected (Figure 3d), in agreement with our findings across tissues from *Canis familiaris* (Seegobin et al., 2018). We also observed a significant increase of DZNT in the supernatant (Figure 3e, *p* < 0.05). Lastly, methylthiolated forms of CTKs were more abundant in the supernatant than the cell pellet, but levels of individual species of 2MeS showed no change in response to LPS treatment (Figure 3g,h).

## 4 DISCUSSION

**FIGURE 5:**
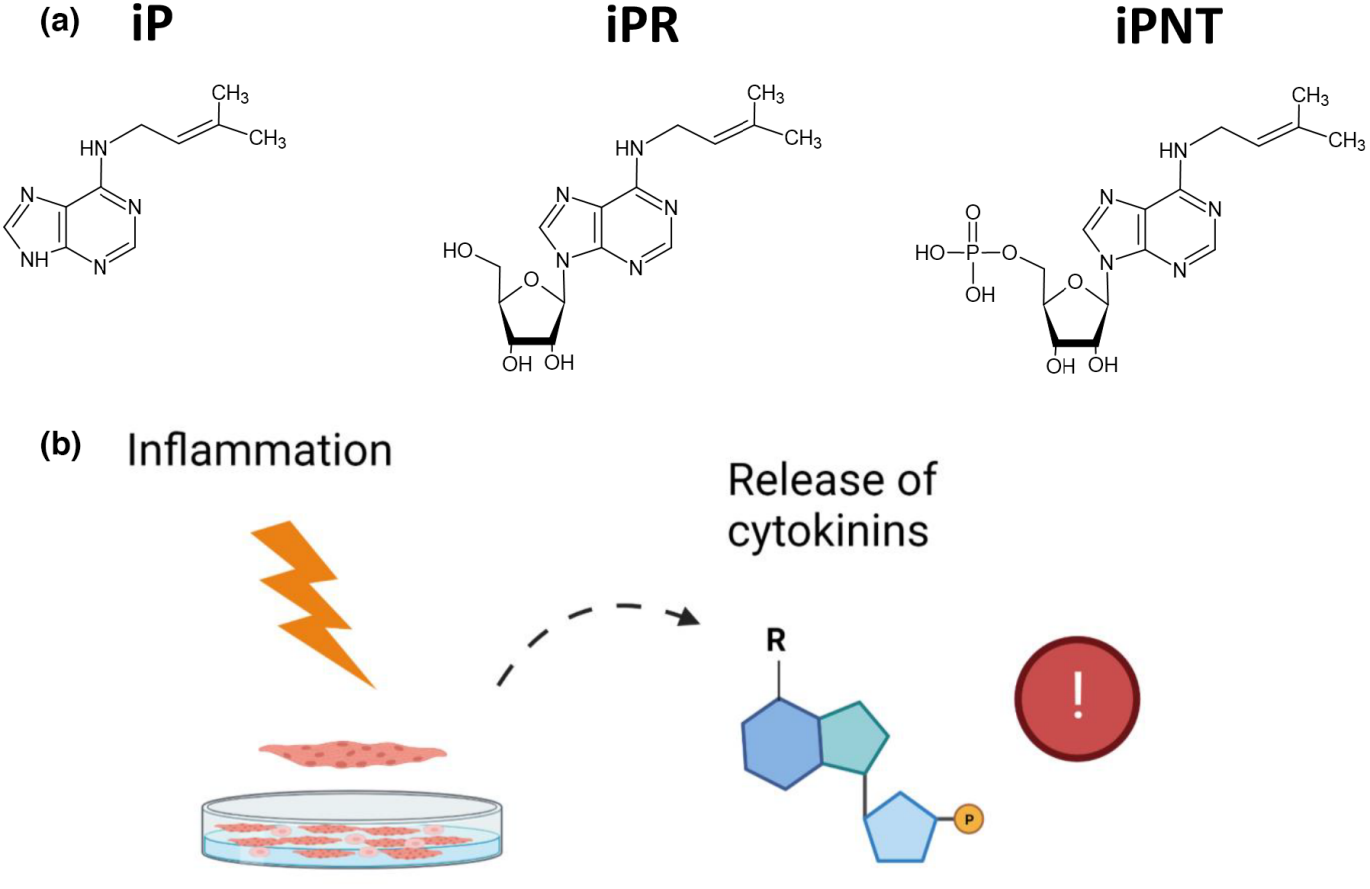
A summary of the chemical structures of isopentenyladenine (iP) and proposed effect of LPS on cytokinin (CTK) release in myogenic cultures. (a) Chemical structures of iP as a free base, riboside (RB), or nucleotide (iPNT). (b) Inflammation (as caused by LPS) induces the release of CTKs which could alert neighboring cells to cellular damage. Image was created with Biorender.com.

We characterized the set of CTK metabolites expressed in stressed cultured mammalian muscle cells, making it a first of its kind study. We show that cellular stress (as induced by pro‐inflammatory LPS) causes an increase in CTKs associated with the tRNA degradation pathway. The increased presence of CTKs in response to LPS is particularly relevant given the importance of purinergic signaling in skeletal muscle (Fulle, 2015; Wang et al., 2023) and the putative role for tRNA‐derived fragments in regulating skeletal muscle homeostasis and stress responses (Liapi et al., 2020). Collectively, iP forms were the most abundant of all CTKs detected both within and outside the cell (Figure 4) and all forms of iP (FB, RB, and NT) were elevated in response to LPS treatment in the extracellular space (chemical structures of iP as a FB, RB, or NT are summarized in Figure 5a). These data suggest that LPS‐mediated myotube cell stress leads to increased production of CTKs. Interestingly, we observed a decrease in IPP, an isoprenoid precursor (Figure 2a) which may be a direct reflection of the use of IPP in the production of CTKs (Aoki et al., 2020).

Recently, extracellular adenosine and adenosine‐5'monophosphate (AMP) were shown to activate AMPK in MuSCs of diabetic mice, resulting in cell cycle arrest (Han et al., 2022). As iP species act as AMP mimetics in epithelial cells to activate AMPK (Pisanti et al., 2014; Ranieri et al., 2018), perhaps iP is serving a similar role in muscle cells to mobilize catabolic processes downstream of AMPK. It is also important to highlight the potential role the CTKs may have in purinergic signaling. In response to cellular damage, development or exercise, extracellular ATP, ADP, AMP, and adenosine are increased (Hellsten et al., 1998). This can in turn alter MuSC (Han et al., 2022) and myofiber activity (Ito et al., 2018). When released to the extracellular space, ATP may also act as damage associated molecular patterns (DAMPs) which can triggers the innate immune response (Panicucci et al., 2020; Patel, 2018). Given the increased presence of extracellular CTKs in response to LPS, we speculate the CTKs may have a role as DAMPs after muscle damage (Figure 5b).

Kinetin, an aromatic CTK, which can promote myogenesis when applied exogenously (Mielcarek & Isalan, 2021), was not detected in our samples in any form (FB, RB, or NT). Kinetin is a naturally occurring metabolite in response to oxidative stress wherein furfuryl groups produced due to hydroxyl radical oxidation of deoxyribose residues react with adenine (Barciszewski et al., 1997). Similar to iP, kinetin species act as adenylate mimetics in the nucleotide salvage pathway resulting in the production of kinetin riboside and kinetin mono‐, di, and triphosphate (Hertz et al., 2013). Exogenous kinetin has been linked to various functions as an antioxidant, to reduce signs of cellular aging and to contribute to the DNA damage response, particularly in Huntington's disease (Bowie et al., 2018). The limit of detection for kinetin in our system is 3.3 pg/mL (Kisiala et al., 2019) which is in the sub‐femtomolar range. It is noteworthy that very few studies ever detect or even attempt to detect endogenous kinetin (Barciszewski et al., 1996; Barciszewski et al., 2000).

This study invokes a number of muscle‐specific and global questions (i.e., the purpose of specific CTK species in cellular function and communication in mammalian cells). Though the biological role of CTK elevation in LPS‐treated myotubes is not yet apparent, given the importance of CTKs in other settings, we hypothesize that changes in CTK profiles are not a passive by‐product but that they have an active role in regulating muscle metabolism and/or manipulating cell behavior within the local muscle microenvironment. For example, CTKs may allow communication between myofibers and other cells of the stem cell niche, such as MuSCs or recruited immune cells. This signaling could be through EVs, as C2C12 derived EVs are enriched in tRNA‐degraded by‐products (Sork et al., 2018). Alternatively, CTKs may have more wide‐reaching effects and function in interorgan communication and endocrine signaling. If so, then profiling serum levels of CTKs from patients with neuromuscular disorders or aged individuals could yield further insight into a number of biological states.

Lastly, our data show that myotubes express 2‐methylthiol (2MeS), dihydrozeatin (DZ), and trans‐Zeatin (tZ) species of CTKs. With respect to DZ and tZ species, this is novel as their detection has not been previously reported in animal tissues or cultures. In plant systems, these CTKs are primarily produced via the de novo biosynthesis pathway. While there have been no reports outlining the presence of a de novo pathway in mammalian systems, it is possible that these species are the product of iP species precursors subject to trans‐hydroxylation. While their biological relevance at endogenous levels remains to be characterized, in exogenous applications zeatin riboside has demonstrated neuroprotective effects via interactions with adenosine A_2A_ receptors (Lee et al., 2012). We also detected these fours methylthiolated CTKs (2MeSiP, 2MeSiPR, 2MeSZ, and 2MeSZR), which is in agreeance with previous findings (Aoki et al., 2019; Seegobin et al., 2018), although their function beyond tRNA has yet to be elucidated.

## 5 LIMITATIONS

Though nucleotides and nucleosides may be released non‐specifically by cell damage or death (Giuliani et al., 2019), we do not yet understand how CTKs are released in mammalian cells. LPS can induce various cellular pathways associated with inflammation, which can activate programs of cell death; however, based on our CCK‐8 assay we did not observe a change in cellular abundance with increasing concentrations of LPS (Figure 2b). An additional consideration is that differentiated C2C12 cells are a mix of myotubes and myogenic reserve cells which have different cellular properties than mature muscles and MuSCs found in vivo. It is possible that different populations of cells contribute and respond to different populations of CTKs species. In the future, CTK levels should be investigated using in vivo models of muscle stress. Lastly, endogenous CTKs were detected in the picomolar range, but most studies using exogenous CTK treatment utilize dosages in the micromolar range (Mielcarek & Isalan, 2021; Pisanti et al., 2014).

## 6 CONCLUSIONS

Our data show for the first time that cultured muscle cells produce and release CTKs in a stress‐responsive manner, suggesting a role for CTKs in intra‐and extracellular signaling of mammalian muscle cells.

## FUNDING INFORMATION

Financial support from the Natural Sciences and Engineering Council of Canada Discovery Grant (NSERC RGPIN‐05436) to RJNE and funding from NSERC and a Trent University Research & Development Grant to SWT (NSERC RGPIN‐1468281).

## CONFLICT OF INTEREST STATEMENT

None to declare.

## ETHICS STATEMENT

No humans or animals were used in this study.

## Supporting information


Table S1.

## Data Availability

The data that support the findings of this study are available from the corresponding author upon reasonable request.
