# Supplementary material for: Profiling of adenine‐derived signaling molecules, cytokinins, in myotubes reveals fluctuations in response to lipopolysaccharide‐induced cell stress
Source: Physiol Rep. 2023 Dec 1;11(23):e15870. doi: 10.14814/phy2.15870 (PMC10691934; doi:10.14814/phy2.15870)
Supplement: Supplementary file 1 — Table S1. [file PHY2-11-e15870-s001.docx]

| **Sample** | **Extracellular CTK Levels (pmol/gFW)** | **p-value** | **Sample** | **Intracellular CTK Levels (pmol/gFW)** | **p-value** |
| --- | --- | --- | --- | --- | --- |
| **2% HS Control 1** | 62.73 |  | **2% HS Control 1** | 79.75 |  |
| **2% HS Control 2** | 74.90 |  | **2% HS Control 2** | 76.53 |  |
| **2% HS Control 3** | 51.65 |  | **2% HS Control 3** | 54.72 |  |
| **Serum Free Control 1** | 54.19 |  | **Serum Free Control 1** | 45.44 |  |
| **Serum Free Control 2** | 81.59 |  | **Serum Free Control 2** | 54.93 |  |
| **Serum Free Control 3** | 118.64 | **0.3354** | **Serum Free Control 3** | 152.33 | **0.7120** |
| **2% HS LPS 1** | 226.45 |  | **2% HS LPS 1** | 103.94 |  |
| **2% HS LPS 2** | 87.15 |  | **2% HS LPS 2** | 63.13 |  |
| **2% HS LPS 3** | 114.84 |  | **2% HS LPS 3** | 53.68 |  |
| **Serum Free LPS 1** | 255.60 |  | **Serum Free LPS 1** | 80.54 |  |
| **Serum Free LPS 2** | 247.43 |  | **Serum Free LPS 2** | 218.30 |  |
| **Serum Free LPS 3** | 304.62 | **0.0520** | **Serum Free LPS 3** | 250.70 | **0.11412** |
